# A defined antigen skin test for the diagnosis of bovine tuberculosis

**DOI:** 10.1126/sciadv.aax4899

**Published:** 2019-07-17

**Authors:** Sreenidhi Srinivasan, Gareth Jones, Maroudam Veerasami, Sabine Steinbach, Thomas Holder, Aboma Zewude, Abebe Fromsa, Gobena Ameni, Laurel Easterling, Douwe Bakker, Nicholas Juleff, Glen Gifford, R. G. Hewinson, H. Martin Vordermeier, Vivek Kapur

**Affiliations:** 1 Department of Animal Sciences, Pennsylvania State University, University Park, PA, USA.; 2 The Huck Institutes, Pennsylvania State University, University Park, PA, USA.; 3 Animal and Plant Health Agency, Surrey, UK.; 4 Cisgen Biotech Discoveries, Chennai, India.; 5 Aklilu Lemma Institute of Pathobiology, Addis Ababa University, Addis Ababa, Ethiopia.; 6 Independent Researcher, Lelystad, Netherlands.; 7 Bill and Melinda Gates Foundation, Seattle, WA, USA.; 8 World Organization for Animal Health, Paris, France.; 9 Centre for Bovine Tuberculosis, Institute for Biological, Environmental and Rural Sciences, University of Aberystwyth, Aberystwyth, UK.

## Abstract

Bovine tuberculosis (bTB) is a major zoonotic disease of cattle that is endemic in much of the world, limiting livestock productivity and representing a global public health threat. Because the standard tuberculin skin test precludes implementation of Bacille Calmette-Guérin (BCG) vaccine–based control programs, we here developed and evaluated a novel peptide-based defined antigen skin test (DST) to diagnose bTB and to differentiate infected from vaccinated animals (DIVA). The results, in laboratory assays and in experimentally or naturally infected animals, demonstrate that the peptide-based DST provides DIVA capability and equal or superior performance over the extant standard tuberculin surveillance test. Together with the ease of chemical synthesis, quality control, and lower burden for regulatory approval compared with recombinant antigens, the results of our studies show that the DST considerably improves a century-old standard and enables the development and implementation of critically needed surveillance and vaccination programs to accelerate bTB control.

## Teaser

A defined antigen skin test has been developed for the reliable diagnosis and vaccine-based control of bovine tuberculosis.

## INTRODUCTION

Tuberculosis, caused by *Mycobacterium tuberculosis* var. tuberculosis, is one of the world’s deadliest infectious diseases, claiming as many as three human lives every minute (*1*–*3*). The closely related *M. tuberculosis* var. bovis (*M. bovis*) is the main cause of tuberculosis in a wide variety of animal hosts, including cattle [bovine tuberculosis (bTB)], and considerably limits livestock productivity (*4*, *5*). bTB represents a serious zoonotic threat and is estimated to cause approximately 10% of the total human tuberculosis cases worldwide (*6*–*8*). While bTB is well controlled in most high-income countries through the implementation of strict test and cull strategies, the disease remains endemic in most low- and middle-income countries, where national control programs have not yet been implemented for socioeconomic reasons, and hence continues to contribute major losses to animal productivity along with human morbidity and mortality (*9*–*12*).

On the basis of an approach initially established more than a century ago, the current standard for diagnosis of bTB in animals measures cell-mediated immune response following an intradermal skin test with the poorly defined and highly variable tuberculin skin test (TST) antigen (*13*, *14*). More recently, an in vitro interferon-γ (IFN-γ) release assay (IGRA) has been introduced as an ancillary test to improve the overall sensitivity of detection of bTB-infected animals (*15*, *16*). The poorly standardized stimulating antigens in the TST [“purified protein derivative” (PPD)] are extracts obtained from the heat-killed cultures of specified strains of mycobacteria grown on glycerol broth (*17*, *18*). For instance, bovine PPD (PPD-B) is derived from an extract of *M. bovis* AN5 strain culture, while avian PPD (PPD-A) is a similarly prepared extract from *M. avium* subsp. *avium* D4ER (*19*). In regions with high exposure to environmental mycobacteria, the difference in increase in skin induration reaction between PPD-B and PPD-A is ascertained using the single intradermal comparative cervical tuberculin test (SICCT) to improve test specificity but is known to reduce assay sensitivity (*13*). Furthermore, in addition to the poor standardization of the PPDs, the presence of cross-reactive antigens between the pathogenic and vaccine strains in the crude whole cell antigen preparation renders the PPD-based TST unable to differentiate infected from Bacille Calmette-Guérin (BCG)–vaccinated animals (DIVA), thereby limiting opportunities for the development of BCG vaccination–based control programs (*20*–*23*). Hence, there is a well-recognized and urgent need to develop defined antigen-based bTB diagnostic assays with DIVA capabilities for use alongside future (vaccination-based) control programs in regions where conventional test and cull strategies are not feasible for socioeconomic reasons (*24*).

Over the past two decades, comparative genomic and transcriptome analyses have identified several specific *M. bovis* antigens with DIVA capability, including ESAT-6, CFP-10, and Rv3615c that are present in field strains of *M. bovis* but are either absent or not immunogenic in the widely used vaccine strain, BCG (*25*, *26*). When used in combination, these antigens have shown promise in both detecting infected animals and differentiating them from those vaccinated with BCG, thereby paving the way for the development of a much-needed defined antigen skin test (DST) with DIVA capability (*27*).

Building on this promising earlier work, the aim of the current study was to develop and evaluate a peptide cocktail of minimum complexity as a defined skin test antigen with DIVA capability that might serve as a reliable, easy to produce, and fit-for-purpose assay for diagnosis of bTB. Last, a considerable advantage of peptide-based reagents is the potential for use under regulatory environments such as in India where genetically modified or recombinant proteins with synthetic tags either are not permitted or face a high threshold for approval. Our investigations suggest that this peptide-based DST not only has equal or superior performance characteristics over the extant standard tuberculin surveillance test but also obviates many of the limitations of the current assays while enabling the development and implementation of critically needed vaccination programs to accelerate bTB control in high prevalence human and bTB settings where it is needed the most.

## RESULTS

### Defined antigens elicit a sensitive and specific in vitro IFN-γ and skin test response in bTB-infected cattle

**Fig. 1:**
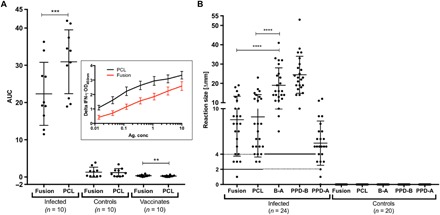
In vitro and skin test responses of fusion protein and PCL. (**A**) Capacity of antigens (Fusion and PCL) to induce in vitro IFN-γ response in PBMCs collected from naturally *M. bovis*–infected cattle (*n* = 10), naïve cattle (*n* = 10), and BCG vaccinates (*n* = 10) was determined. The antigens were used at titrated dose concentrations, and AUC is plotted. The horizontal line provides the mean (±SD), and the statistical difference between the responses was determined by using two-tailed paired *t* test (**P < 0.01; ***P < 0.001). The background-corrected optical density (OD) values of the Fusion and PCL at each titrated concentration tested are shown in the inset. The PPD-B–induced IGRA responses of these animals tested in (A) are shown in fig. S1. (**B**) Skin test responses for Fusion, PCL, and PPD(B-A) were measured at 72 hours after injection in cattle experimentally infected with *M. bovis* (*n* = 24) and naïve controls *(n* = 20). Results are expressed as the difference in skin thickness (in millimeters) between the pre- and post-skin test readings, with the horizontal line providing the median [±95% confidence interval (CI)]. The statistical difference between the responses was determined using analysis of variance (ANOVA) (****P < 0.0001). The solid horizontal lines at 2 and 4 mm are the cutoffs used for Fusion and PCL, and PPD(B-A) and PPD-B, respectively.

A comparison of the performance of a peptide cocktail composed of 40-mer peptides covering the sequences of ESAT-6, CFP-10, and Rv3615c with a 20-residue overlap [peptide cocktail–long (PCL)] and a recombinant fusion protein of the same three antigens was undertaken using IGRAs, with peripheral blood mononuclear cells (PBMCs) isolated from naturally *M. bovis*–infected cattle, naïve controls, and BCG vaccinates. The IGRAs were performed to establish a dose-response relationship, and the results were expressed as area under the curves (AUC; Fig. 1A). The data demonstrated that PCL induced significantly stronger in vitro IFN-γ responses in infected cattle compared to the fusion protein (*P* = 0.0004; Fig. 1A). In contrast, both PCL and the fusion protein induced minimal, if any, IFN-γ responses in PBMCs from control or vaccinated animals, together suggesting potential utility of defined antigens in in vitro IFN-γ assays to specifically identify *M. bovis*–infected cattle (Fig. 1A). These data suggested that both DST reagents may also be applied to the blood test commonly used as ancillary test to skin testing in cattle.

The performances of PCL and the fusion protein as defined skin test antigens were next assessed in animals experimentally infected with *M. bovis* (*n* = 24). The results showed that when applying the established strict DST interpretation criteria of 2 mm or more increase in skin induration reaction [using Receiver Operating Characteristic (ROC) analyses to set this cutoff point], PCL was able to correctly classify all of the infected animals as positive, while the fusion protein identified 23 of 24, although this difference was not statistically significant (*P* = 0.8401; Fig. 1B). In contrast, neither PCL nor fusion protein induced measurable skin induration responses in a control group of naïve animals (*n* = 20). Together, these results suggest that while PCL performs better than the fusion protein in IGRAs and equivalently in skin tests, they are both able to accurately classify infected from uninfected animals with high apparent sensitivity and specificity.

### A subset of peptides drives most of the observed immune responsiveness in infected cattle

**Fig. 2:**
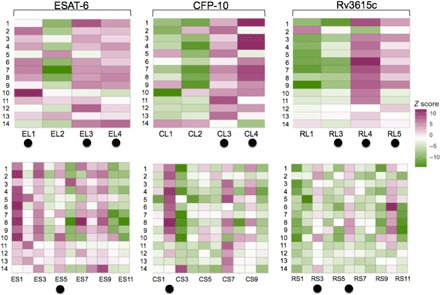
Heat map representing the IFN-γ response elicited by the individual long (top) and short (bottom) peptides of ESAT-6, CFP-10, and Rv3615c. ELISpot/FluoroSpot assays were performed using PBMCs isolated from naturally *M. bovis*–infected cattle (*n* = 14). *Z* scores of individual peptide response are mapped. On the basis of overall peptide responder frequencies, a new peptide cocktail was formulated, PC-1. The peptides constituting PC-1 are marked with closed circles. Note: RL2 could not be synthesized due to technical difficulties, and RS5 was included as part of PCL to cover the gap in the overlap left by the absence of RL2.

Next, we performed extensive peptide mapping experiments to identify immune-dominant peptides within PCL in an attempt to develop a peptide cocktail of reduced complexity, containing only the most dominant and major histocompatibility complex (MHC)–promiscuous recognized peptides. In addition, this provided an opportunity to identify individual 40-mer peptides that might be susceptible, for example, to inappropriate processing or other modifications that might lead to loss of immune recognition. To mitigate this potential risk, we mapped a set of shorter overlapping peptides described in a previous study (*28*), with the aim of identifying better responding shorter substitute peptides covering the same epitopic region. Individual peptides from PCL and of a corresponding set of overlapping short, 16– to 20–amino acid–long peptides were synthesized and screened for their ability to elicit IFN-γ responses in Enzyme-linked immune absorbent spot (ELISpot) assays using PBMCs isolated from naturally *M. bovis*–infected cattle (*n =* 14). The results show that most of the observed immune responsiveness within PCL and the shorter peptides are driven by only a subset of peptides recognized promiscuously by almost every animal tested (Fig. 2). This result suggested that further refinement of the compositional complexity of PCL was possible. The data also suggest that some epitopes when contained within the long peptides were not as well recognized than when they were represented on the shorter peptides (Fig. 2). For example, a strong epitope within ESAT-6 is localized within the short peptide ES5. Consequently, the expectation was that EL2 would be strong and promiscuously recognized as well. However, as Fig. 2 shows, peptide EL2 was poorly and infrequently recognized. Other similar examples also occurred with peptide CL1, which is poorly recognized, while the short peptide CS2, whose sequence is contained in CL1, is strongly recognized. Similarly, RL1 and RL3 were poorly recognized but their poor recognition could be compensated by the short peptides RS3 and RS6 (Fig. 2).

### Optimized peptide cocktail PC-1 displays comparable sensitivity and specificity as PCL

**Fig. 3:**
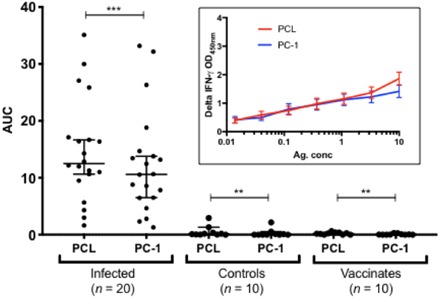
In vitro responses of PCL and PC-1. Capacity of PCL and PC-1 to induce in vitro IFN-γ responses in PBMCs collected from naturally *M. bovis*–infected cattle (*n* = 20), BCG vaccinates (*n* = 10), and controls (*n* = 10) was evaluated. The antigens were used at titrated dose concentrations, and AUC is plotted. The horizontal line provides the median (±95% CI), and the statistical difference between the responses for reactors and controls was determined using the Wilcoxon matched-pairs signed-rank test (***P < 0.001; **P < 0.01), while two-tailed *t* test was used for vaccinates (**P < 0.01). There is no significant difference between the responses induced by PCL and PC-1 in infected animals. The background-corrected OD values of PCL and PC-1 at each titrated concentration tested are shown in the inset, where PCL induced a significantly stronger response than PC-1 only at the highest titrated dose concentration of 10 μg/ml. PPD-B–induced IGRA responses of these animals are shown in fig. S2B.

On the basis of the strength of immune response induced by individual peptide antigens and the sequence overlaps between PCL and shorter peptides, we assembled a cocktail [peptide cocktail 1 (PC-1)] representing a combination of promiscuously recognized long and short peptides (table S1). The capability of PC-1 to induce an IFN-γ response was assessed with IGRAs performed on cryopreserved PBMCs isolated from known naturally infected cattle (*n =* 20), naïve controls (*n* = 10), and BCG vaccinates (*n* = 10). The results showed that PCL induced a significantly stronger response in infected animals compared to PC-1 (*P* < 0.001), although this was driven by a stronger response elicited by PCL at only the highest titrated dose concentration (10 μg/ml), but not others (Fig. 3, inset). This high concentration also induced nonspecific IGRA responses in some of the naïve animals and is therefore not a peptide concentration that is diagnostically relevant due to its relative lack of specificity. If this concentration was disregarded in calculating the AUCs, PCL and PC-1 performed identically (fig. S2A). Only limited, if any, IGRA responses based on evaluation of AUC values were induced by either cocktail when they were tested with PBMCs from naïve or BCG-vaccinated animals (Fig. 3).

### PCL demonstrates superior performance compared with conventional PPD-based assays in naturally infected cattle in an endemic country setting

**Fig. 4:**
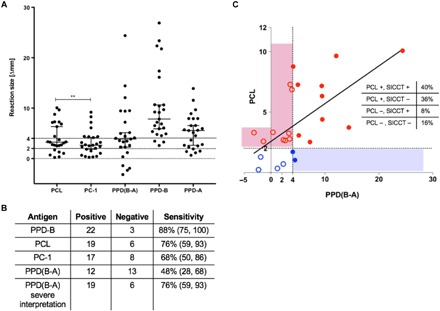
Skin test response to peptide cocktails in field reactors. (**A**) Peptide cocktails, PCL and PC-1, alongside PPD-A and PPD-B, were tested in naturally infected animals identified in Ethiopia (*n* = 25). Results are expressed as the difference in skin thickness (in millimeters) between the pre- and post-skin test readings, with the horizontal line providing the median (±95% CI). Positivity is defined as ≥2 mm for PCL and PC-1, and ≥4 mm for PPD(B-A) and PPD-B (denoted by the solid lines). The statistical difference between PCL and PC-1 responses was determined by using two-tailed *t* test (**P < 0.01). (**B**) The relative sensitivity of the peptide cocktails, SIT, and SICCT was calculated. (**C**) Animals represented by open red circles are positive by PCL only, closed blue circles are positive by PPD(B-A) only, open blue circles are negative on both, and closed red circles are positive on both. The peptide cocktail, PCL, identified nine (36%) presumed infected animals that were not detected by the standard SICCT.

To assess and compare the performance characteristics of PCL as a defined skin test antigen with current PPD-based standards in crossbred cattle in endemic country settings, we tested PCL and PC-1 alongside PPD-A and PPD-B in a naturally infected herd of Zebu cattle in Ethiopia (*n* = 25). The results showed that PPD-B, PCL, and PC-1 identified 22, 19, and 17 animals, respectively, of 25 animals in this herd as infected (Fig. 4, A and B). PCL, PC-1, and PPD-B identified a greater number of infected animals compared to the SICCT, which only correctly classified 12 of 25 animals. The disappointing performance of the SICCT in this herd is consistent with the observations that a high burden of exposure to environmental mycobacteria, as evidenced by the increase in skin thickness induced by PPD-A (Fig. 4A), is a well-known SICCT confounder reducing its sensitivity (*13*, *29*, *30*). In the herd tested, 10 of 22 PPD-B–positive animals tested SICCT negative due to a stronger response to PPD-A than PPD-B (Fig. 4A). Furthermore, of the 13 animals that tested negative in the SICCT, 9 tested positive to PCL (Fig. 4C, open red circles), demonstrating that the majority (69%) of naturally infected animals missed by SICCT were detected by PCL (Fig. 4C). PCL also matched the number of positives obtained with the high sensitivity interpretation of SICCT (severe interpretation). Together, the suggestion of superior performance of PCL and PC-1 compared to SICCT presented in this study in a high-bTB burden herd with concurrent high levels of sensitization to environmental mycobacteria is encouraging and prioritizes them for further evaluations to accurately define their performance under field conditions.

## DISCUSSION

Comparative genomic, transcriptomic, and proteomic analyses have identified several promising *M. bovis* antigens with DIVA capability including the highly immunogenic proteins ESAT-6, CFP-10, and Rv3615c that are present in *M. bovis* but either absent or not immunogenic in all BCG vaccine strains (*26*, *27*, *31*–*34*). Used in combination as defined antigens, these proteins have shown considerable promise in detecting *M. bovis*–infected animals and in differentiating them from those vaccinated with BCG in both skin test and laboratory assays. Extending these data with recombinant protein-based DST, the results of the current study show that a fusion protein containing these three antigens was able to induce specific skin test responses to detect infected cattle (Fig. 1). However, peptide-based reagents may have potential for use even in regions with high regulatory barriers for in vivo use of recombinant-derived products in food-producing animals, and our work described here using synthetic peptides was undertaken to overcome the limitations on the use of recombinant proteins in such regulatory environments. Synthetic peptide-based DST for bTB diagnosis has recently shown promise as an alternative to recombinant proteins (*27*, *31*, *32*, *35*). However, these peptide cocktails were composed of large and complex sets of short overlapping 16- to 20-mer peptides with limited commercial use as a skin test reagent due to cost challenges associated with manufacture and quality assurance of complex peptide mixtures. To address these challenges, our current studies show that a less complex cocktail of 40-mer-long overlapping peptides (PCL) perform superior to the recombinant fusion protein in IGRA and equivalent in inducing skin responses in infected animals while maintaining the high specificity in naïve or BCG-vaccinated animals.

We attempted to reduce the complexity of the peptide cocktail by mapping the immunodominant peptides within PCL as well as a shorter set of overlapping peptides using ELISpot assays (Fig. 2). The findings suggest that while most of the PCL peptides were strongly recognized, a smaller subset appeared poorly antigenic for reasons that are not fully understood but may include steric hindrance in fitting of the peptides in the MHC binding groove or inappropriate processing (*36*, *37*). For example, CL1 appeared to be poorly recognized in ELISpot assays, while the short peptide CS2, whose sequence is located within CL1, was recognized promiscuously (Fig. 2). In contrast, a number of the long peptides, such as CL3 and CL4 on the C terminus of CFP-10, elicited a far stronger response than short peptides CS6 to CS10 that cover the same region of the sequence. These observations highlight the opportunity to optimize and refine the cocktail composition by inclusion of combinations of immune-dominant long and short peptides that together elicited both broad and promiscuous responses.

To explore this possibility, a refined combination of peptide antigens was designed (PC-1, table S1) and evaluated in vitro and in animal studies. The results showed a similar in vitro IFN-γ response of both PC-1 and PCL in reactor cattle (Fig. 3). Because studies performed under controlled experimental conditions often overestimate diagnostic test performance in comparison with field studies, we next sought to assess the performance of the peptide cocktails under field conditions in a bTB endemic region. The results show that there is no significant difference in the proportion of animals testing positive to PC-1 and PCL in skin tests (Fig. 4B). Together, these data provide compelling evidence of the potential to reduce the complexity of the peptide cocktail even while further enhancing and optimizing performance characteristics by selecting the optimal combination of dominant long and short peptides for inclusion as a defined skin test antigen.

To assess performance characteristics in endemic country settings, the relative sensitivity of the PCL and PC-1 peptide cocktails was assessed in a group of 25 naturally infected reactor crossbred cattle in Ethiopia. The results confirm that the relative diagnostic sensitivity of the synthetic peptide cocktails (PCL and PC-1) did not significantly differ from that of the Single Intradermal Test (SIT) (PPD-B) (Fig. 4B). In contrast, at 48%, the relative sensitivity of the PPD(B-A) response was 40% lower than that observed for PPD-B alone, suggesting that in these animals the relative sensitivity of the widely used and The World Organization for Animal Health/Office International des Epizooties (OIE)–prescribed SICCT assay for endemic regions was significantly (*P* = 0.005; Fisher exact; two-tailed) lower than that of the SIT. While this finding is not altogether surprising, given the likely high burden of environmental mycobacteria in Ethiopia and other countries in which bTB remains endemic, it underscores the primary dilemma of tuberculin-based skin testing in bTB endemic countries with high or unknown burden of environmental mycobacteria: The use of PPD-B alone as in with the SIT often results in high rates of false-positive results due to sensitization with environmental mycobacteria, and conversely, the comparative (SICCT) test has the potential for a high false-negative rate due to the masking of the PPD-B responses in animals sensitized with environmental mycobacteria (*13*, *29*). In contrast, by using defined antigens, the DST has the potential to obviate many of the limitations of traditional tuberculin-based tests and also provides an opportunity to obtain diagnostic sensitivities equivalent to that of the SIT and specificities equal or exceeding the SICCT, all within a single injection site.

The results of this and other studies (*27*, *35*) suggest that the use of defined antigens provides considerably lower amplitude of skin reactions in reactor animals (in this study: 8 ± 1.5 mm with PCL as compared with an average of 19.5 ± 3.5 mm with PPD-B) without compromising sensitivity. Furthermore, because the DST obviates the need for administration of PPD-A to improve test specificity, animals that are exposed to environmental mycobacteria that lack or do not express the DST antigens remain unreactive, providing a considerable technical advantage as well as animal welfare benefits when considering routine surveillance of animals in regions with high exposure to environmental mycobacteria.

While the DST shows considerable promise as a fit-for-purpose test for routine surveillance and diagnosis of bTB-infected animals, given the lack of concordance between the different tests used for bTB diagnosis under field conditions (Fig. 4 and fig. S4), it will be important for future studies to establish the true status of infection in field reactor animals to obtain robust estimates of test sensitivity or specificity. For instance, although all of the cattle included in our experiment in Ethiopia were presumably *M. bovis*–infected animals because they were part of a heavily infected herd and at the very least were highly exposed to *M. bovis* in their environment, their true infection status remains unknown without conducting a thorough postmortem examination, which is a challenge even in high-income country settings. Because of the lack of an accurate reference antemortem test for infection, it is not surprising that trials for validation of tuberculosis diagnostics are difficult to perform under field conditions, particularly in endemic country settings (*38*, *39*). To address this issue, large-scale surveillance programs in endemic country settings combined with powerful statistical approaches including maximum likelihood estimation and Bayesian inferences will be required for accurate estimates of DST or TST performance and are planned. We note that although the 2-mm cutoff for DST applied in the current study was established using ROC analyses, this will need to be reassessed as additional data from field trials with the DST become available to ensure optimal balance between sensitivity and specificity in relevant geographies to best meet local needs.

An important goal remaining in the field of bTB diagnostics is to improve upon the performance of tuberculin-based tests. Our current study provides strong evidence that defined peptide-based DST may represent promising alternatives to the traditional tuberculin-based assays for bTB diagnosis in field settings, and additionally provides the ability to differentiate infected from BCG-vaccinated animals, thereby overcoming a major hurdle for the implementation of BCG cattle vaccination programs as a key component of disease control in low- and middle-income countries.

Promising results of preliminary safety trials (not shown) with DST in *Bos taurus* ssp. *indicus* crossbred cattle under Good Laboratory Practice (GLP) conditions in India with repeat and overdosing experiments suggest that the DST is safe. Future efforts are needed to assess the performance characteristics of DST in larger cohorts of infected, control, and BCG-vaccinated animals under field conditions to satisfy OIE standards for assay validation, as well as provide the scientific basis for implementation of the DST in national bTB surveillance and control programs and critically needed tools to accelerate control of bTB in the 21st century.

## MATERIALS AND METHODS

### Antigens and peptides

Commercial preparations of bovine tuberculin (PPD-B) and avian tuberculin (PPD-A, Thermo Fisher Scientific) were used to stimulate PBMCs at a final concentration of 300 and 250 IU/ml, respectively, as per kit instructions. Two sets of peptides [PCL, 40-mer length with a 20-residue overlap; PCS (peptide cocktail–short), 16 to 20 mers with an 8- to 12-residue overlap] were chemically synthesized to a minimal purity of 98 and 85%, respectively. The identity was confirmed by mass spectrometry (see table S1 for amino acid sequences). A histidine-tagged fusion recombinant protein of ESAT-6, CFP-10, and Rv3615c, expressed in *Escherichia coli*, was purified by nickel affinity chromatography (Lionex Ltd., Braunschweig, Germany).

### Animals

For the in vitro experiments conducted at Animal and Plant Health Agency (APHA), archived PBMCs from the following groups of cattle (*B. taurus*) were used: (i) naturally *M. bovis*–infected cattle originating from U.K. herds known to have bTB (natural infection was confirmed by postmortem and/or culture analysis), (ii) noninfected control cattle originating from U.K. herds in the low risk area that were officially tuberculosis free for more than 5 years, and (iii) BCG vaccinates consisting of control cattle as described in (ii) that were subsequently vaccinated with BCG Danish Statens Serum Institut (SSI) (equivalent to five human doses). PBMCs from 8 weeks after vaccination were used. For in vivo testing of the peptide cocktails, the following groups of cattle were used: (i) experimentally *M. bovis*–infected cattle consisting of male calves experimentally infected with approximately 10,000 colony-forming units of a field strain of *M. bovis* (AF2122/97) via the endobronchial route (skin tests were performed 5 weeks after infection, and infection was confirmed by postmortem and/or culture analysis), and (ii) noninfected control cattle as described above. Last, to determine performance characteristics of the DST in endemic country settings, in vivo sensitivity of the peptide cocktails was also assessed in 25 adult crossbred cattle (Holstein Friesian X Zebu) in Ethiopia. These cattle were initially recruited from the Holeta National Dairy Research Center of the Ethiopian Institute of Agricultural Research as they were positive for bTB and gave strong positive responses to both SICCT and IGRA. All cattle experiments were performed in accordance with animal ethics and biosafety protocols approved by the Aklilu Lemma Institute of Pathobiology Review Board (reference no. ALIPB IBR/007/2011/2018). Animal procedures for studies conducted at APHA were approved by the APHA Animal Welfare and Ethical Review Body and in Ethiopia by the Addis Ababa University Ethics Review Committee.

### Skin test procedures

PPD-A and PPD-B were administered in a 0.1-ml volume as per the manufacturer’s recommendations, while peptides were administered as a cocktail containing 10 μg of each peptide (0.1-ml final volume) based on previously established dose titration experiments performed by skin testing cattle (*27*). Skin thicknesses were measured by the same operator before and at 72 hours after administration, and the difference in skin thickness (in millimeters) between the pre- and post-skin test readings was recorded as per OIE-prescribed guidelines (*19*).

### In vitro stimulation of PBMCs

The PBMC preparation was performed following the “overlay” method using tubes without frit and cryopreserved. Thawing of cryopreserved cells was performed as quickly as possible in a water bath at 37°C. Upon thawing, appropriate volume of complete medium [RPMI 1640 containing 2 mM GlutaMax, 25 mM Hepes, 0.1 mM NEAA (nonessential amino acids), 5 × 10^−5^ M β-mercaptoethanol, penicillin (100 U/ml), streptomycin (100 μg/ml) (Gibco Life Technologies, UK), and 10% fetal calf serum (Sigma-Aldrich, UK)] was added in a drop-by-drop manner and centrifuged at 350*g* for 10 min at room temperature. The supernatant was carefully discarded, and the cell pellet was gently loosened, following which it was resuspended in an appropriate volume of fresh complete medium depending on the required concentration for the assay. Cells were counted using a hemocytometer and incubated with the antigens for in vitro stimulation. For ELISpot/FluoroSpot, following an incubation period of ~20 hours, the spots were developed as described below. For BOVIGAM, the incubation period was 3 to 5 days, after which the plates were centrifuged at 350*g* for 10 min at room temperature and the supernatant was carefully harvested.

### IFN-γ enzyme-linked immunosorbent assay

IFN-γ concentrations in PBMC culture supernatants were determined using the commercially available BOVIGAM enzyme-linked immunosorbent assay–based kits (Thermo Fisher Scientific, USA). Results were initially expressed as the optical density at 450 nm (OD_450_) for cultures stimulated with antigen minus the OD_450_ for cultures without antigen (i.e., ΔOD_450_). The results from antigen dose titration curves allowed AUC values to be calculated using Prism 7 (GraphPad Software, La Jolla, CA) software.

### IFN-γ ELISpot/FluoroSpot assay

Stimulation of PBMCs for these assays was performed as previously described (*40*). The production of IFN-γ by PBMCs was detected using either (i) a secondary biotinylated antibody followed by incubation with streptavidin-linked horseradish peroxidase (Bovine IFN-γ ELISpot kit; Mabtech, Stockholm, Sweden), with visualization using an AEC chromogen kit (Sigma, St. Louis, MO) and spot-forming units counted using an AID ELISPOT reader and ELISpot 4.0 software (Autoimmun Diagnostika, Germany), or (ii) a secondary BAM-conjugated anti-bovine IFN-γ antibody (clone MT307, Mabtech) followed by incubation with a fluorophore-labeled anti–BAM-490 (Mabtech) antibody, with visualization using a fluorescence enhancer (Mabtech). Spot-forming units were counted using an ELISpot/FluoroSpot reader system (iSpot Spectrum, AID, Germany) with software version 7.0. The average response elicited in an individual animal to a particular protein was calculated as a way of normalizing within each animal. The statistical difference between these average responses and the individual peptide responses in all animals was determined using *z* scores. *Z* score helped determine SDs of the raw scores of individual peptides (number of IFN-γ–positive spots) from the average response per animal, and the results were mapped as shown in Fig. 2. For example, ELISPOT reader counted 99, 74, 268, and 296 spots for EL1, EL2, EL3, and EL4, respectively, for animal no. 1 (mean response elicited by animal no. 1 for ESAT-6 is 184.25). *Z* scores were found to be −2.4, −10.9, 7.2, and 3.2 for EL1, EL2, EL3, and EL4, respectively. A schematic representation of the approach is presented in fig. S4 and shows that the greater the z score, the stronger the individual peptide response relative to the average response elicited by the animal.

### Statistical analysis

All statistical analyses were performed using Prism 7 (GraphPad Software, La Jolla, CA).

## Supplementary Material

http://advances.sciencemag.org/cgi/content/full/5/7/eaax4899/DC1

Download PDF

## SUPPLEMENTARY MATERIALS

Supplementary material for this article is available at http://advances.sciencemag.org/cgi/content/full/5/7/eaax4899/DC1 Table S1. All short and long peptides used in the study are listed and sequences are provided. Fig. S1. PPD-B responses in the IGRA. Fig. S2. Comparison of performance of PCL with PC-1 in IGRA. Fig. S3. Skin test responses in field reactors. Fig. S4. Schematic representation of the approach to identify the dominant peptides based on overall responder frequency for the peptide cocktails.
