# Supplementary material for: A defined antigen skin test for the diagnosis of bovine tuberculosis
Source: Sci Adv. 2019 Jul 17;5(7):eaax4899. doi: 10.1126/sciadv.aax4899 (PMC6636981; doi:10.1126/sciadv.aax4899)
Supplement: http://advances.sciencemag.org/cgi/content/full/5/7/eaax4899/DC1 [file supp_5_7_eaax4899__index.html]

Science Advances | Science AdvancesAAASSearchScience AdvancesMenu

## Supplementary Materials

**This PDF file includes:**

- Table S1. All short and long peptides used in the study are listed and sequences are provided.
- Fig. S1. PPD-B responses in the IGRA.
- Fig. S2. Comparison of performance of PCL with PC-1 in IGRA.
- Fig. S3. Skin test responses in field reactors.
- Fig. S4. Schematic representation of the approach to identify the dominant peptides based on overall responder frequency for the peptide cocktails.

Download PDF

**Files in this Data Supplement:**

- Adobe PDF - aax4899\_SM.pdf
