# Supplementary material for: A defined antigen skin test for the diagnosis of bovine tuberculosis
Source: Sci Adv. 2019 Jul 17;5(7):eaax4899. doi: 10.1126/sciadv.aax4899 (PMC6636981; doi:10.1126/sciadv.aax4899)
Supplement: Download PDF [file aax4899_SM.pdf]

Science Advances
AAAS

advances.sciencemag.org/cgi/content/full/5/7/eaax4899/DC1

Supplementary Materials for

A defined antigen skin test for the diagnosis of bovine tuberculosis

Sreenidhi Srinivasan, Gareth Jones, Maroudam Veerasami, Sabine Steinbach, Thomas Holder, Aboma Zewude, Abebe Fromsa, Gobena Ameni, Laurel Easterling, Douwe Bakker, Nicholas Juleff, Glen Gifford, R. G. Hewinson, H. Martin Vordermeier, Vivek Kapur*

*Corresponding author. Email: vkapur@psu.edu

Published 17 July 2019, *Sci. Adv.* **5**, eaax4899 (2019)
DOI: 10.1126/sciadv.aax4899

This PDF file includes:

Table S1. All short and long peptides used in the study are listed and sequences are provided.
Fig. S1. PPD-B responses in the IGRA.
Fig. S2. Comparison of performance of PCL with PC-1 in IGRA.
Fig. S3. Skin test responses in field reactors.
Fig. S4. Schematic representation of the approach to identify the dominant peptides based on overall responder frequency for the peptide cocktails.

Supplementary Materials

Supplementary Table:

Table S1. All short and long peptides used in the study are listed and sequences are provided. Peptides EL1 to EL4, CL1 to CL4, RL1 to RL5 and RS5 constitute PCL. Note: RL2 could not be synthesized due to technical difficulties and RS5 was included as part of PCL to cover the gap in the overlap left by the absence of RL2. All peptides constituting PC-1 are marked with an asterisk.

**Nomenclature**	**Peptide sequence**
EL1 *	MTEQQWNFAGIEAAASAIQGNVTSIHSLLDEGKQSLTKLA
EL2	NVTSIHSLLDEGKQSLTKLAAAWGGSGSEAYQGVQQKWDA
EL3 *	AAWGGSGSEAYQGVQQKWDATATELNNALQNLARTISEA G
EL4 *	TATELNNALQNLARTISEAGQAMASTEGNVTGMFA
CL1	MAEMKTDAATLAQEAGNFERISGDLKTQIDQVESTAGSLQ
CL2	ISGDLKTQIDQVESTAGSLQGQWRGAAGTAAQAAVVRFQE
CL3 *	GQWRGAAGTAAQAAVVRFQEAANKQKQELDEISTNIRQAG
CL4 *	AANKQKQELDEISTNIRQAGVQYSRADEEQQQALSSQMGF
RL1	MTENLTVQPERLGVLASHHDNAAVDASSGVEAAAGLGESV
RL3 *	AITHGPYCSQFNDTLNVYLTAHNALGSSLHTAGVDLAKSL
RL4 *	AHNALGSSLHTAGVDLAKSLRIAAKIYSEADEAWRKAIDG
RL5 *	RIAAKIYSEADEAWRKAIDGLFT
ES1	MTEQQWNFAGIEAAAS
ES2	AGIEAAASAIQGNVTS
ES3	AIQGNVTSIHSLLDEG

ES4	IHSLLDEGKQSLTKLA
ES5 *	KQSLTKLAAAWGGSGS
ES6	AAWGGSGSEAYQGVQQ
ES7	EAYQGVQQKWDATATE
ES8	KWDATATELNNALQNL
ES9	LNNALQNLARTISEAG
ES10	ARTISEAGQAMASTEG
ES11	QAMASTEGNVTGMFA
CS1	MAEMKTDAATLAQEAGNF
CS2 *	QEAGNFERISGDLKTQ
CS3	ERISGDLKTQIDQVESTA
CS4	IDQVESTAGSLQGQWRG
CS5	GSLQGQWRGAAGTAAQAA
CS6	AGTAAQAAVVRFQEAANK
CS7	VVRFQEAANKQKQELDEI
CS8	QKQELDEISTNIRQAGVQYS
CS9	NIRQAGVQYSRADEEQQQ
CS10	RADEEQQQALSSQMGF
RS1	MTENLTVQPERLGVLASHHD
RS2	PERLGVLASHHDNAAVDASS
RS3 *	SHHDNAAVDASSGVEAAAGL
RS4	DASSGVEAAAGLGESVAITH
RS5	AAGLGESVAITHGPYCSQFN
RS6 *	AITHGPYCSQFNDTLNVYLT

RS7	SQFNDTLNVYLTAHNALGSS
RS8	VYLTAHNALGSSLHTAGVDL
RS9	LGSSLHTAGVDLAKSLRIAA
RS10	GVDLAKSLRIAAKIYSEADE
RS11	RIAAKIYSEADEAWRKAIDG

Supplementary Figures:

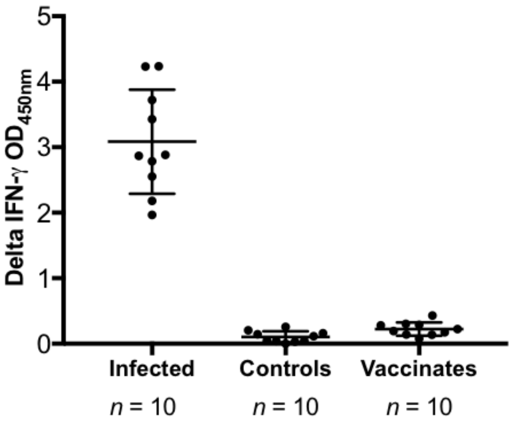

Fig. S1. PPD-B responses in the IGRA. This was performed using PBMCs collected from naturally *M. bovis*-infected cattle (*n* = 10), naïve cattle (*n* = 10) and BCG vaccinates (*n* = 10) are shown. The horizontal line provides the mean (± standard deviation), and results are expressed as the background-corrected OD_{450}.

Fig. S2. Comparison of performance of PCL with PC-1 in IGRA.

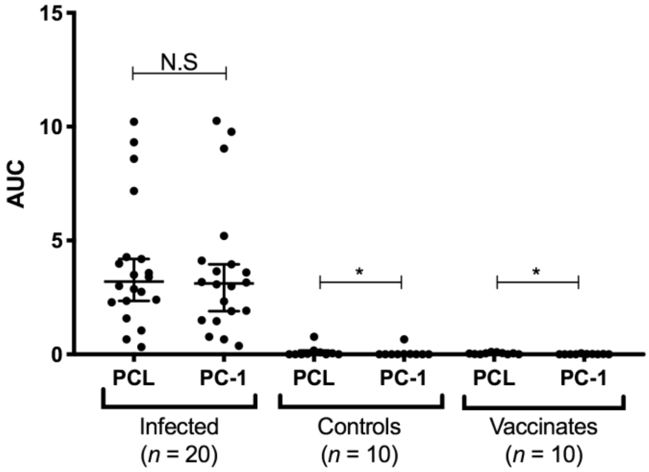

Fig. S2A. The capacity of PCL and PC-1 to induce *in vitro* IFN-*γ* responses. This was performed in PBMCs collected from naturally *M. bovis*-infected cattle (*n* = 20), BCG vaccinates (*n* = 10) and controls (*n* = 10) was evaluated. The antigens were used at titrated dose concentrations and Area under the curve (AUC) is plotted disregarding the highest concentration (10 *μ*g/ml). The horizontal line provides the median (± 95% CI), and the statistical difference between the responses for reactors and controls was determined using the Wilcoxon matched-pairs signed rank test (***, $P < 0.001$), while two-tailed t test was used for vaccinates (*, $P < 0.05$).

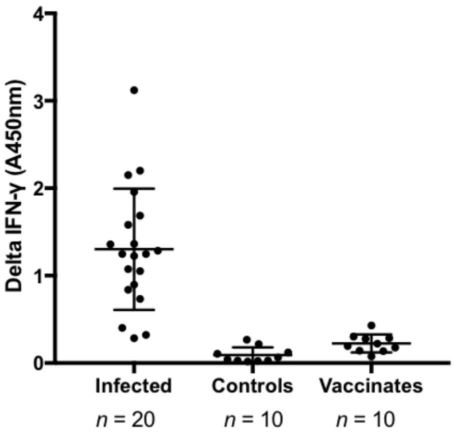

Fig. S2B. PPD-B responses in the IFN-gamma release assay. This was performed using PBMCs collected from naturally *M. bovis*-infected cattle (*n* = 20), naïve cattle (*n* = 10) and BCG vaccinates (*n* = 10) are shown. The horizontal line provides the mean (± standard deviation), and results are expressed as the background-corrected OD_{450}.

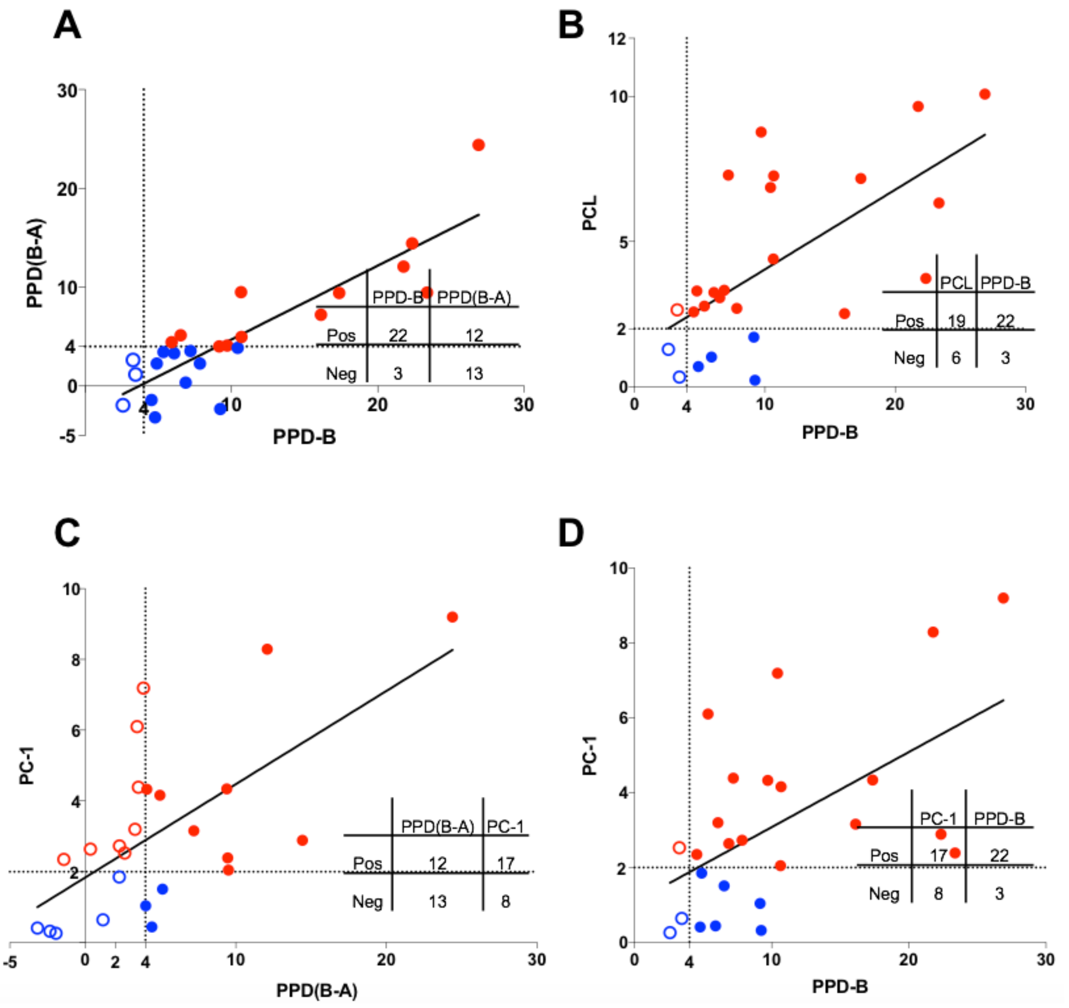

Fig. S3. Skin test responses in field reactors. The closed red circles represent the animals that were identified as positive to both the antigens, open blue circles denote the animals that were negative to both tests, closed blue circles are animals positive to the antigen on the x-axis and negative to the antigen on the y-axis, and open red circles are animals negative to the antigen on the x-axis and positive to the antigen on the y-axis in any given plot. The r^2 values are as follows: (A) 0.75, (B) 0.42, (C) 0.41 and (D) 0.32.

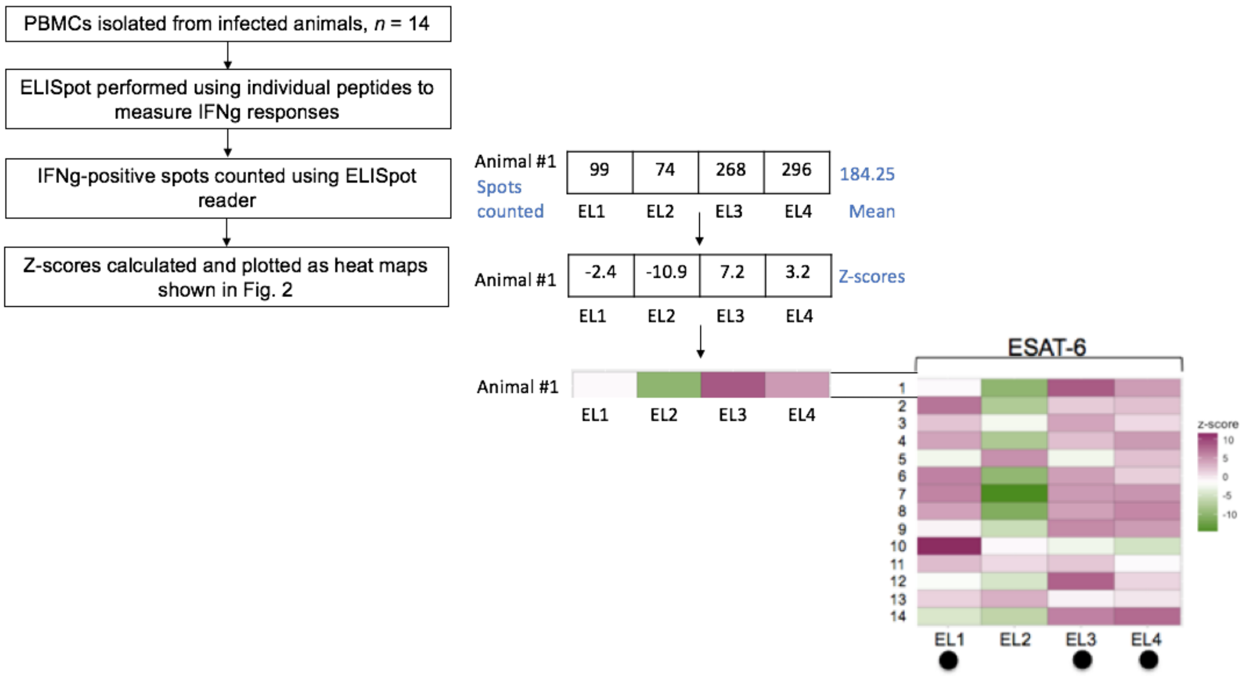

Fig. S4. Schematic representation of the approach to identify the dominant peptides based on overall responder frequency for the peptide cocktails. In brief, PMBCs were isolated from 14 infected reactor animals, ELISpot assays were performed to measure IFN-g responses for each individual peptide and Z-scores calculated and plotted as heatmaps. An example is shown for ESAT-6. Similar plots were derived for CFP-10 and Rv3615c and consolidated as presented in Fig. 2.
